# Clinical features and prognosis of polypoidal choroidal vasculopathy with different morphologies of branching vascular network on optical coherence tomography angiography

**DOI:** 10.1038/s41598-021-97340-1

**Published:** 2021-09-08

**Authors:** Shang-Te Ma, Chu-Hsuan Huang, Yun-Chia Chang, Tso-Ting Lai, Yi-Ting Hsieh, Tzyy-Chang Ho, Chung-May Yang, Cheng-Guo Cheng, Chang-Hao Yang

**Affiliations:** 1 Department of Ophthalmology, Taipei Medical University-Shuang Ho Hospital, New Taipei City, Taiwan; 2 Department of Ophthalmology, National Taiwan University Hospital, Taipei, Taiwan; 3 Department of Ophthalmology, Cathay General Hospital, Taipei, Taiwan; 4 Department of Ophthalmology, Shin Kong Wu Ho-Su Memorial Hospital, Taipei, Taiwan; 5 Department of Ophthalmology, National Taiwan University College of Medicine, Taipei, Taiwan

**Keywords:** Retinal diseases, Eye diseases, Macular degeneration

## Abstract

This study highlights the clinical features and treatment response of polypoidal choroidal vasculopathy (PCV) among three different branching vascular network (BVN) morphologies in optical coherence tomography angiography (OCTA), and further correlates the BVN features with those under fluorescent angiography (FA) and indocyanine green angiography (ICGA). In total, we reviewed 70 eyes with PCV followed up for > 12 months. OCTA, ICGA and FA images were obtained at baseline and post-treatments. BVN was assessed using OCTA and divided into three types by a previously described BVN classification: type 1 (trunk), type 2 (glomeruli), and type 3 (stick). At baseline, type 1 BVN had the poorest vision and thinnest subfoveal choroidal thickness (SFCT), whereas type 3 had the best vision and thickest SFCT. The aforementioned trend sustained after treatments. Each BVN morphology in OCTA showed typical features in FA + ICGA and encompassed significant correlation (*p* = 0.004). In conclusion, OCTA is an innovative imaging tool for the detection and classification of BVN in PCV. Furthermore, OCTA has advantages of being noninvasive and free of systemic toxicities. The BVN can be divided into three types based on morphological characteristics in OCTA, which play crucial roles in clinical presentations and treatment outcomes.

## Introduction

Polypoidal choroidal vasculopathy (PCV) and neovascular age-related macular degeneration (nAMD) have certain similarities, but PCV has some clinical characteristics distinct from classic choroidal neovascularisation^1–3^. Choroidal neovascularisation with polypoidal structures and branching vascular networks (BVNs) are the hallmarks of PCV, and leakage from the pathological vasculature may cause recurrent serosanguinous detachment of sensory retina and pigment epithelium, which further deteriorates vision^4–6^.

Previously, indocyanine green angiography (ICGA) was used to diagnose and assess the outcomes of PCV lesions^7,8^. For instance, polyps with the grape cluster configuration^9–11^, large polyps^12^, and polyp pulsation^13^ may account for worse prognostic visual outcomes. Owing to the diversity of clinical presentation, several categorising methods were proposed to differentiate PCV lesions into clinically significant types. Kawamura et al. divided PCV lesions into two subtypes based on ICGA findings, which was according to the presence of feeding origins. The two subtypes were different in terms of choroidal thickness and the number of detectable polyps^14^. Furthermore, Inoue et al. classified PCV lesions into “polypoidal choroidal vasculopathy” and “polypoidal choroidal neovascularisation” (PCNV) based on OCT images showing the presence of pachyvessels in the choroid or typical age-related macular degeneration (AMD) features^15^. Similarly, Coscas et al.^16^ investigated both ICGA and OCT images and proposed two PCV types: idiopathic PCV and polyps associated with nAMD. In addition to structural differences, Coscas et al. observed that the visual acuity was significantly better in the idiopathic PCV group. However, no further implications were stated regarding visual prognosis in the aforementioned studies.

Tan et al. proposed an innovative classification system of dividing PCV lesions into three BVN subtypes based on ICGA and fluorescent angiography (FA) images (types A, B, and C), which successfully predicted visual prognosis at 5 years^17^. Nevertheless, both ICGA and FA involve intravenous injection of dye for image retrieval. Severe anaphylactic reactions, although rare but detrimental, have been sporadically reported with the dye injection^18–20^. Conversely, OCT for choroidoretinal pathology assessment improved patients’ safety and satisfaction revolutionarily^21^. OCT advancement further aided the visualisation of chorioretinal microvascular structures and provides en face imaging of high quality^22,23^. With the detection of red blood cell movement through decorrelation motion contrast between repeated scans, optical coherence tomography angiography (OCTA) can retrieve vascular images noninvasively^24–26^.

Huang et al. reported a novel BVN morphologic classification system with clinical relevance for treatment outcomes for PCV lesions that used OCTA^27^. However, clinical implications for long-term visual prognosis of PCV revealed through OCTA were not provided yet. In this study, the authors aimed to analyse the correlation between different BVN morphologies and biomarkers in PCV lesions through OCTA and FA + ICGA. Furthermore, the discrepancies in the demographic factors and prognosis between different BVN morphological types were re-validated and investigated.

## Methods

This retrospective cohort study was performed in National Taiwan University Hospital (NTUH, Taipei, Taiwan, R.O.C.). The study was approved by the International Review Board of the National Taiwan University Hospital (20170803RINC) and was performed in accordance with the tenets of the Declaration of Helsinki. Owing to the nature of a tertiary referral and a clinical study centre in NTUH, the informed consents were obtained from the patients upon every visit and treatment episode.

### Study design and image analysis

The authors recruited patients with PCV and followed them up for > 12 months in NTUH from January 2015 to December 2017. According to the predetermined study protocols, all included patients underwent OCTA, ICGA, and FA examination and received the combination therapy of photodynamic therapy (PDT) with a single anti-vascular endothelial growth factor (VEGF) agent (bevacizumab, ranibizumab, or aflibercept). OCTA images were retrieved using AngioVue (RTVue XR Avanti, Optovue Inc., Fremont, CA, USA), whereas ICGA + FA images were captured using SPECTRALIS HRA + OCT (Heidelberg Engineering, Heidelberg, Germany). The treatment protocol for the combination therapy of PDT has been reported previously^28^. The patients received an intravitreal injection of a single anti-VEGF agent followed by a PDT session. Furthermore, anti-VEGF intravitreal injection was subsequently administered monthly for the next 2 months. For patients who deferred PDT and only received anti-VEGF therapy, we omitted them from further study and statistics according to the study protocols and submitted IRB.

The patients with polyps occupying more than 3 × 3 mm area from the central macula, macular scarring, or choroidal neovascularisation (such as proliferative diabetic retinopathy and myopic choroidal neovascularisation) caused by factors other than PCV were excluded. The authors recorded age, sex, and PDT re-treatment status of all the enrolled patients. Moreover, the best-corrected visual acuity (BCVA) and imaging features in OCTA and ICGA+FA were recorded at baseline and 3, 6, and 12 months post-treatment to assess the treatment response. The BVN area were calculated by AngioVue OCTA system and were further analysed. The assessed biomarkers on structural OCT included subfoveal choroidal thickness (SFCT), central retinal thickness (CRT), subretinal fluid (SRF), retinal pigment epithelial detachment (RPED), and intraretinal cyst (IRC). All OCTA and ICGA+FA images were interpreted by two independent retinal specialists (S.-T.M. and C.-H.H.) to classify the PCV lesions. All disparities in interpretation between the two reviewers were arbitrated by another retinal specialist (C.-H.Y.).

Furthermore, we performed re-treatment and rescue therapy under appropriate clinical scenarios depending on clinicians’ personal preferences in accordance to the PCV treatment consensus in Taiwan^29^. PCV reactivation was considered if new onset of vision loss ≥ 1 line or equivalent, and at least one additional criterion as follows was met: (1) subretinal or intraretinal fluid, (2) RPED, (3) subretinal or sub-retinal pigment epithelial (sub-RPE) haemorrhage, or (4) obvious fluorescent leakage. If the reactivation was confirmed by two independent specialists, the patients received further re-treatment or rescue therapy^30^.

### BVN morphological classifications

In the present study, the authors complied with the BVN morphological classification system presented by Huang et al.^27^ Three distinct BVN patterns were identified in PCV lesions through OCTA and categorised as follows: Type 1 (trunk form), characterised by a major vascular trunk that further radiates into smaller vessel calibre pointing to the periphery; Type 2 (glomeruli form), characterised by prominent anastomoses of vascular calibre; and Type 3 (stick form), characterised by localised, fine, and thin neovascular network without identifiable main feeders or anastomoses. In this manuscript, we named this as NTUH morphologic classification.

As previously reported, Tan et al. divided PCV lesions into three subtypes (types A, B and C) based on BVN characteristics in ICGA and FA images^17^. Type A PCV was characterised by BVN with interconnecting vessels in ICGA without an obvious feeding origin, whereas the other two types of PCV consisted of distinct BVN and feeding vessels on ICGA, without (type B) or with (type C) detectable leakage through FA. Considering that both classification systems focused on the features of BVN in PCV lesions, the authors thus tried to disclosure the associations in both classification systems, if any.

### Statistical analysis

The categorised data and the correlation of BVN features in OCTA and ICGA+FA were evaluated using chi-square test or Fisher exact test. Continuous data were analysed using analysis of variance (ANOVA) test, and Bonferroni corrections were conducted for post hoc tests. SPSS version 22.0 (IBM Corp., Armonk, NY, USA) and EXCEL 2011 (Microsoft, Inc. Redmond, Washington, USA) were used for statistical analyses. A *p* value of < 0.05 was regarded statistically significant.

## Results

The authors found 78 eyes of 78 patients with PCV between 2015 and 2017, and only 8 patients underwent monotherapy of anti-VEGF and were thus omitted from further analysis according to the predetermined study protocols. Distinct BVN morphological patterns were detected in all the eyes after manual adjustment of segmentation lines on OCTA.

### Demographics and the correlation of imaging features on OCTA and ICGA + FA

Of the 70 patients, the average age of the patients was 67.9 ± 7.5 years, and 46 (65.7%) patients were men. All the enrolled patients were treatment-naïve at baseline. In terms of BVN morphology, 30 eyes (42.9%) were type 1 (trunk form), 22 eyes (31.4%) were type 2 (glomeruli form), and 18 (25.7%) were type 3 (stick form) according to the definition of Huang et al. The Cohen’s Kappa Coefficient of agreement between two readers was 0.910.

The baseline demographic features are summarised in Table 1. The average BCVA at baseline in logarithm of the minimum angle of resolution (LogMAR) was 0.75 ± 0.57, and 34 (48.6%) patients needed re-treatment or rescue therapy within 1 year of the first PDT regimen. Men were more predominant (95.5%) in the type 2 BVN group than in the other two groups (*p* = 0.007). The mean BCVA at baseline was significantly worse in the type 1 BVN group than in the other two groups (*p* = 0.036). Regarding OCT structural biomarkers, the SFCT was significantly thick in the type 3 BVN group and thin in the type 1 BVN group (*p* < 0.001). Moreover, the BVN encompassed area was the largest in type 1 BVN (trunk form) subgroup (*p* = 0.024) and foveal involvement was seen in all the 30 patients. No statistical discrepancies were observed between the aforementioned three BVN types for CRT, SRF, RPED, or IRC. The multivariate linear regression analysis showed that vision at baseline was significantly related to the BVN morphological type revealed through OCTA and the existence of IRC (*p* = 0.029 and 0.040, respectively).

**Table 1:** Baseline demographic data and clinical features of PCV patients according to the BVN morphologic classification system.

NTUH BVN morphological classification					
	Total (n = 70)	Trunk (n = 30)	Glomeruli (n = 22)	Stick (n = 18)	*p* value
Age (years)	67.9 ± 7.5	68.5 ± 8.0	70.1 ± 7.7	65.3 ± 6.7	0.137^†^
Sex (M:F)	46:24	16:14	21:1	9:9	0.007*^‡^
BCVA(logMAR)	0.75 ± 0.57	0.98 ± 0.66	0.67 ± 0.49	0.53 ± 0.43	0.036*^†§^
PDT retreatment (%, n)	48.6 (34)	50.0 (15)	59.1 (13)	33.3 (6)	0.263^**‡**^
OCT-SFCT (μm)	251.5 ± 80.4	214.9 ± 63.1	249.1 ± 54.2	311.3 ± 96.3	< 0.001*^†¶^
OCT-CRT (μm)	282.3 ± 85.1	266.5 ± 66.8	308.6 ± 105.2	273.9 ± 80.9	0.272^†^
OCTA-BVN area (mm^2^)	0.624 ± 0.343	0.751 ± 0.415	0.643 ± 0.441	0.392 ± 0.239	**0.024**^†^
OCT-SRF (%, n)	72.9 (51)	73.3 (22)	77.3 (17)	66.7 (12)	0.561^**‡**^
OCT-RPED (%, n)	97.1 (68)	100 (30)	95.5 (21)	94.4 (17)	0.450^**‡**^
OCT-IRC (%, n)	41.4 (29)	50.0 (15)	36.4 (8)	33.3 (6)	0.894^**‡**^

Abbreviations: *M* male, *F* female, *BCVA* best-corrected visual acuity, *PDT* photodynamic therapy, *SFCT* subfoveal choroidal thickness, *CRT* central retinal thickness, *SRF* subretinal fluid, *RPED* retinal pigment epithelial detachment, *IRC* intraretinal cyst, *PCV* polypoidal choroidal vasculopathy, *BVN* branching vascular network, *NTUH* National Taiwan University Hospital, *OCT* optical coherence tomography.

Bold values indicate *p* < 0.05 and were considered significant. ^†^One-way analysis of variance and Bonferroni test for post hoc evaluation if *p* < 0.05. ^**‡**^Chi-square or Fisher’s exact test. ^§^Post hoc test: *p* = 0.042, between types 1 and 3 and post hoc test: *p* = 0.034, between types 1 and 2. ^¶^Post hoc test: *p* = 0.009, between types 1 and 3.

In the present study, we found that the NTUH morphological classification of BVN through OCTA and the features of BVN in ICGA + FA proposed by Tan et al. had significant correlations (Table 2, *p* = 0.004, chi-square test).

**Table 2:** Correlation of BVN morphology detected using OCTA and ICGA + FA in PCV patients.

ICGA + FA morphology (n)	OCTA morphology (n)			
	Type 1	Type 2	Type 3	
	Trunk (30)	Glomeruli (22)	Stick (18)	
Type A (13)	3 (23.1%)	3 (23.1%)	**7 (53.8%)**	***p* = 0.004**^†^
Type B (12)	2 (16.7%)	**8 (66.7%)**	2 (16.7%)	
Type C (45)	**25 (55.6%)**	11 (24.4%)	9 (20.0%)	

Abbreviations: *OCTA* optical coherence tomography angiography, *ICGA* indocyanine green angiography, *FA* fluorescent angiography, *PCV* polypoidal choroidal vasculopathy.

^†^Chi-square test. Values in bold indicate *p* < 0.05 and were considered statistically significant.

**Figure 1:**
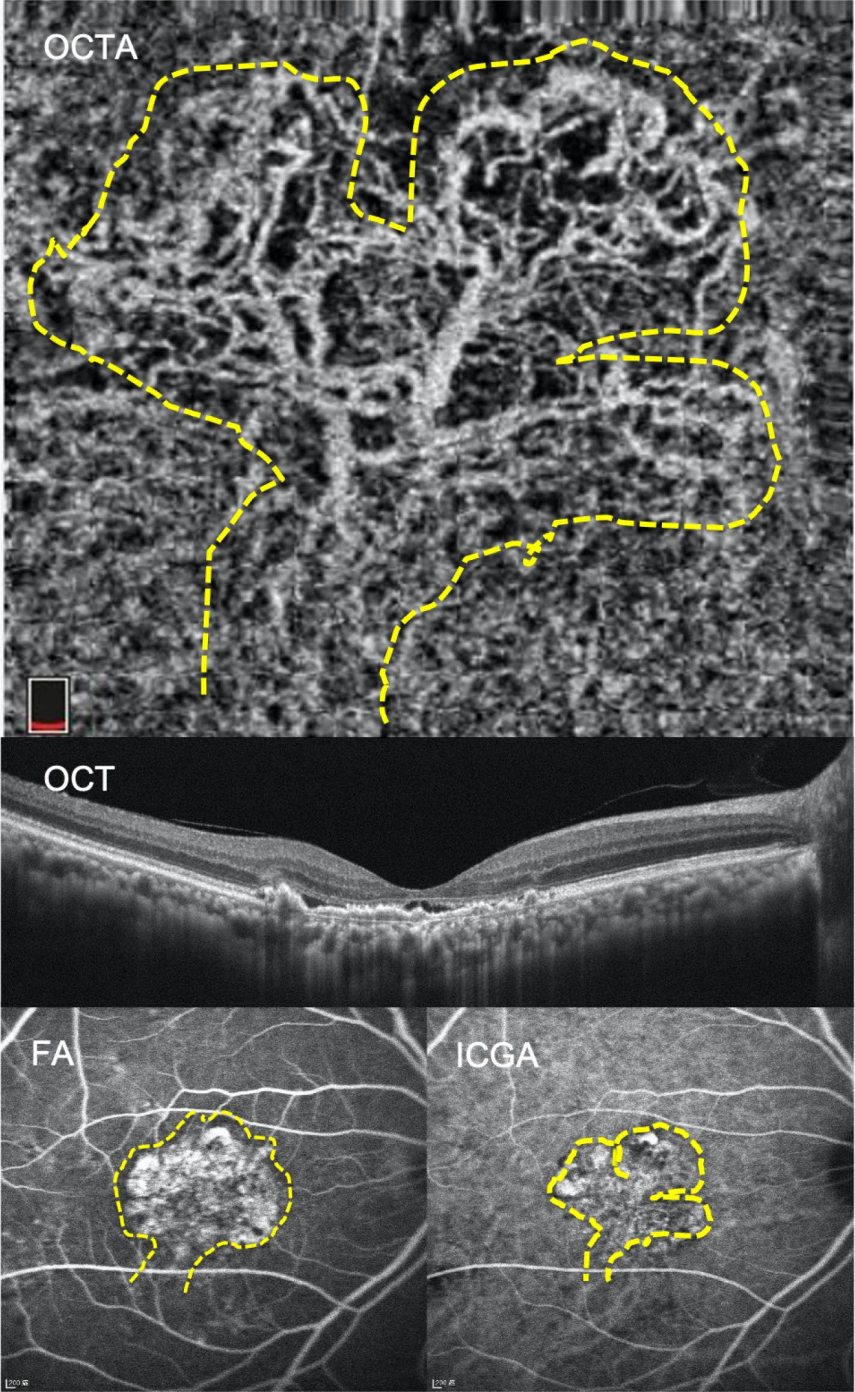
The branching vascular network (BVN) morphology revealed through optical coherence tomography angiography (OCTA) had significant correlation with polypoidal choroidal vasculopathy (PCV) characteristics revealed through indocyanine green angiography (ICGA) and fluorescent angiography (FA). A 72-year-old female patient had type 1 BVN and type C PCV in the right eye. Baseline best corrected visual acuity was 20/800. The OCTA image showed major vascular trunk with further branching of BVN (trunk form, highlighted by dashed line). The subfoveal choroidal thickness was 200 μm. ICGA showed a cluster of polypoid lesions and BVN with a definite feeding origin; obvious leakage was also detected through FA. Multiple drusenoid deposits and retinal pigment epithelial detachment existed. *BVN* branching vascular network, *PCV* polypoidal choroidal vasculopathy, *BCVA* best-corrected visual acuity, *OCTA* optical coherence tomography angiography, *OCT* optical coherence tomography, *FA* fluorescent angiography, *ICGA* indocyanine green angiography.

**Figure 2:**
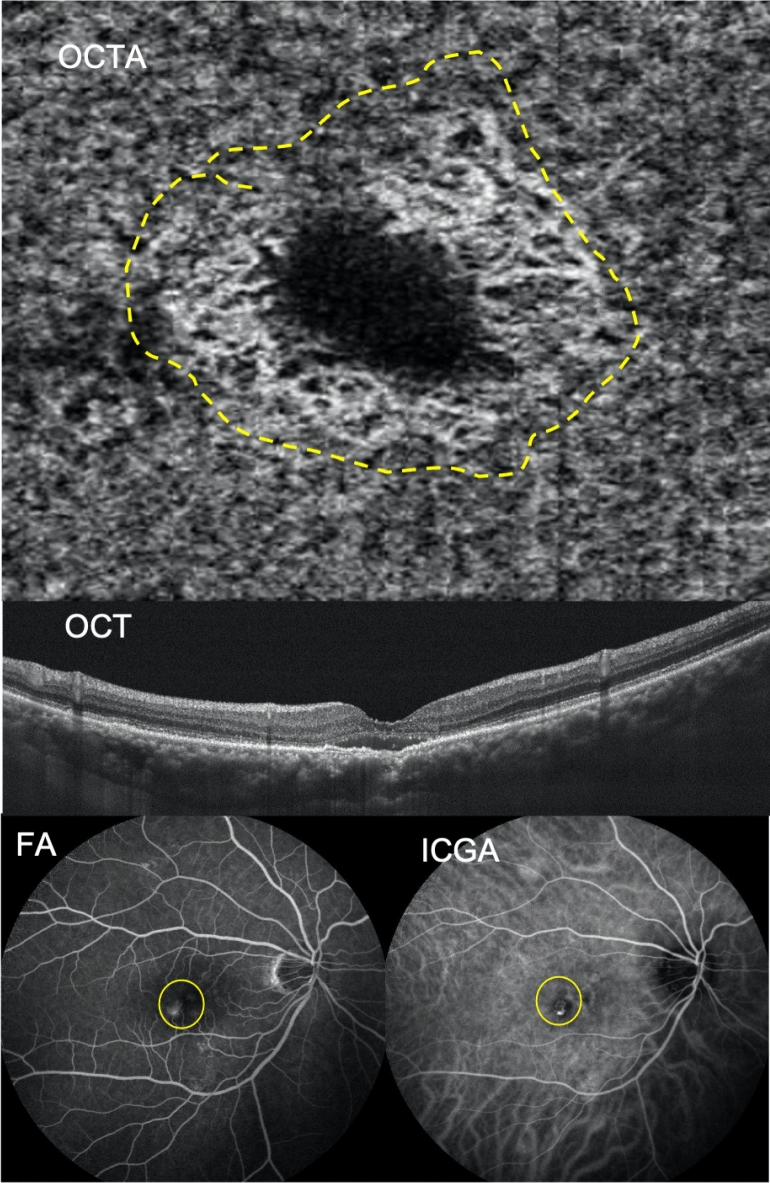
A 67-year-old male patient had type 2 BVN and type B PCV in the right eye. Baseline BCVA was 20/200. The OCTA image retrieved by AngioVue (RTVue XR Avanti, Optovue Inc., Fremont, CA, USA) showed prominent anastamoses of BVN which resembled nephrons (glomeruli form, highlighted by the dashed line), and RPED with SRF also existed. The SFCT was 275 μm. Both FA and ICGA images showed a cluster of BVN with definite feeding origin, but late leakage was not detected by FA (circle). *BVN* branching vascular network, *PCV* polypoidal choroidal vasculopathy, *BCVA* best-corrected visual acuity, *FA* fluorescent angiography, *ICGA* indocyanine green angiography, *OCTA* optical coherence tomography angiography, RPED: retinal pigment epithelial detachment, *SRF* subretinal fluid, *SFCT* subfoveal choroidal thickness, *OCT* optical coherence tomography.

**Figure 3:**
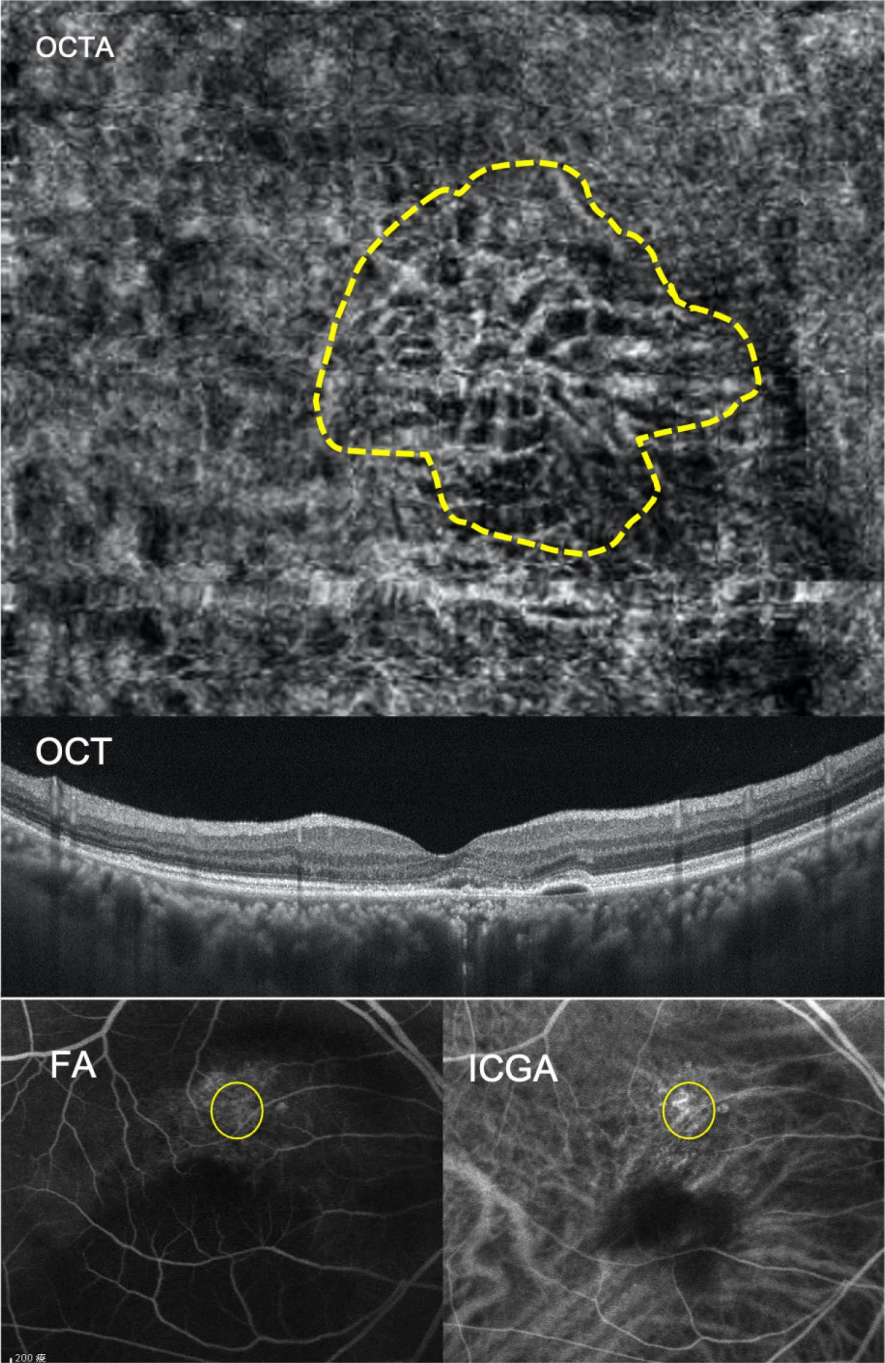
A 65-year-old male patient with type 3 BVN and type C BVN in the right eye. Baseline BCVA was 20/80. The OCTA image retrieved by AngioVue (RTVue XR Avanti, Optovue Inc., Fremont, CA, USA) disclosed small, tangled calibre of choroidal vessel without significant anastomoses (stick form, highlighted by dashed line) and perifoveal RPED with some subretinal fluid. The SFCT was approximately 350 μm. FA and ICGA images showed an intercollecting vessel without an obvious feeding origin (circle). *BVN* branching vascular network, *PCV* polypoidal choroidal vasculopathy, *BCVA* best-corrected visual acuity, *FA* fluorescent angiography, *ICGA* indocyanine green angiography, *OCTA* optical coherence tomography angiography, *RPED* retinal pigment epithelial detachment, *SFCT* subfoveal choroidal thickness.

For the majority of patients, types 1, 2, and 3 BVN in OCTA correlated with types C, B, and A in ICGA and FA, respectively (Figs. 1, 2, 3). The BVN types did not convert from one type to another after treatment in our studied cases.

### Comparisons of visual outcomes and OCT structural biomarkers

**Figure 4:**
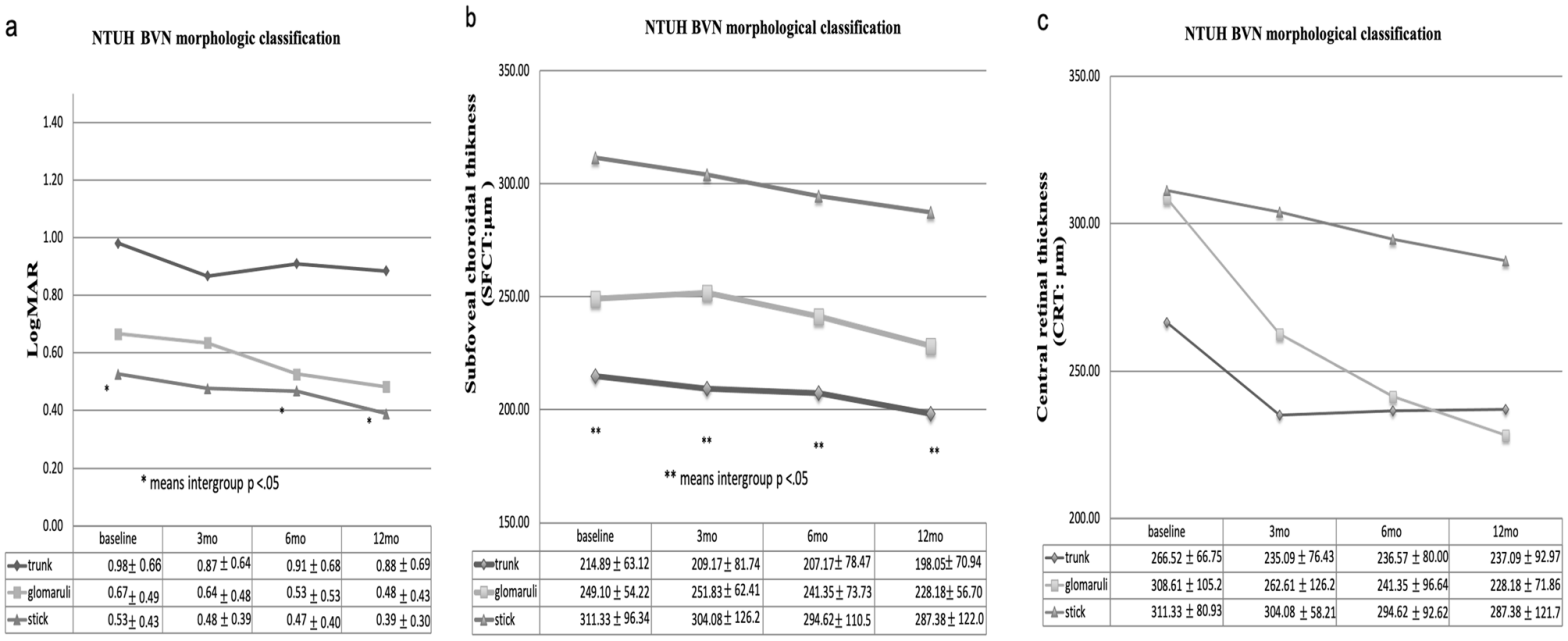
Comparison of changes in (**a**) BCVA in logMAR, (**b**) subfoveal choroidal thickness (SFCT), and (**c**) central retinal thickness (CRT) at different time points between the BVN types in PCV lesions: type 1: trunk form, type 2: glomeruli form, and type 3: stick form. The figure was depicted by EXCEL 2011 (Microsoft, Inc. Redmond, Washington, USA). *BCVA* best-corrected visual acuity, *SFCT* subfoveal choroidal thickness, *CRT* central retinal thickness, *BVN* branching vascular network, *PCV* polypoidal choroidal vasculopathy, *NTUH* National Taiwan University Hospital.

In the present cohort, all the enrolled patients were followed up for > 12 months after the first treatment. Overall, the BCVA improved from 0.75 ± 0.57 to 0.66 ± 0.60 (logMAR, *p* < 0.001) at 12 months post-treatment. The comparisons of the BCVA between the three BVN subtypes at each time point are depicted in Fig. 4a. The BCVA significantly improved in type 2 (from 0.67 ± 0.49 to 0.48 ± 0.42 at 12 months post-treatment, logMAR, *p* < 0.001) and type 3 (from 0.53 ± 0.43 to 0.39 ± 0.30 at 12 months post-treatment, logMAR, *p* = 0.012). By contrast, the BCVA showed limited improvement in type 1 BVN (from 0.98 ± 0.66 to 0.88 ± 0.69 at 12 months post-treatment, logMAR, *p* = 0.01). The patients with type 3 BVN had the most advantageous BCVA among the three types not only at baseline but also at 12 months post-treatment (*p* = 0.005). After the first treatment, 34 (48.6%) patients needed re-treatment within 1 year follow-up period (Table 1). The PDT re-treatment rate was non-significantly higher in patients with type 1 and type 2 BVN (50.0% and 59.1%, respectively, *p* = 0.263).

Among the biomarkers on structural OCT, the SFCT showed a common trend among the different BVN types at baseline and after treatment. The SFCT ultimately remained the lowest, intermediate, and highest in types 1, 2, and 3, respectively, at each time point (*p* < 0.05 for all, Fig. 4b). Furthermore, the SFCT decreased significantly in each BVN type at 12 months after treatment compared with that at baseline (*p* < 0.05 for each type). Conversely, the CRT did not significantly differ among the three morphologic types at each time point (Fig. 4c). Nevertheless, the CRT after PDT continued to decline after treatment in all BVN types, and the findings were significant in patients with type 2 BVN (3 months post-treatment compared with baseline, *p* = 0.026) and type 3 BVN (all time points compared with baseline, *p* < 0.01 for all).

The pattern of chorioretinal fluid compartments, namely SRF, RPED, and IRC, in different PCV types was thoroughly investigated. Of the 70 eyes studied at baseline, we found 51 (72.9%), 68 (97.1%), and 29 (41.4%) eyes with SRF, RPED, and IRC, respectively. At baseline, SRF was the most prevalent in type 2 BVN (77.3%), whereas RPED and IRC were the most prevalent in type 1 BVN (RPED and IRC: 100% and 50%, respectively). After treatment, the presence of SRF and IRC decreased universally, but no significant difference was observed among the three types at each time point. Multivariate linear regression analysis for visual outcomes at 12 months post-treatment is shown in Table 3. Overall, BVN morphology and the presence of IRC significantly affected the initial visual performance and final visual prognosis.

**Table 3:** Linear regression analysis for prognostic factors correlated to the final visual prognosis after the first episode of PDT at 12 months post-treatment.

	Regression coefficient	*p* Value
Sex	0.070	0.585
BVN morphology on OCTA	− 0.332	**0.008**
Baseline CRT	− 0.020	0.877
Baseline SFCT	− 0.159	0.213
Baseline IRC	− 0.033	0.823
Baseline SRF	0.092	0.473
Baseline PED	0.060	0.640
CRT at month 12	− 0.063	0.635
SFCT at month 12	− 0.236	0.064
IRC at month 12	0.493	**0.002**
SRF at month 12	0.104	0.419
RPED at month 12	0.019	0.884

*PDT* photodynamic therapy, *BVN* branching vascular network, *OCTA* optical coherence tomography angiography, *CRT* central retinal thickness, *SFCT* subfoveal choroidal thickness, *IRC* intraretinal cyst, *SRF* subretinal fluid, *RPED* retinal pigment epithelial detachment.

Bold values indicate *p* < 0.05 and were considered statistically significant.

## Discussion

In the present study, the authors reappraised the BVN morphological classification system with an extended observation period and successfully demonstrated that PCV outcomes can be prognosticated using OCTA^27^. We not only extended the follow-up duration to 12 months post-treatment but also investigated the demographic factors, imaging characteristics on OCTA, initial visual acuity, and re-treatment rate of patients with each BVN type. In addition, the correlations of BVN imaging features between OCTA and ICGA + FA were validated.

Owing to more experience with manual segmentation using OCTA, the detection rate of BVN was 100% in our study. Many studies have demonstrated that BVN detection using OCTA might be more sensitive than detection through traditional ICGA^31,32^. Recent efforts were made to diagnose PCV lesions without ICGA. Chaikitmongkol et al.^33^ observed that the existence of more than two of four imaging criteria (RPED on colour fundus photography, peaked RPED, notched RPED, and hyperreflective ring with RPED in OCT) could successfully diagnose PCV lesions with satisfactory sensitivity and specificity. In addition, the Asia–Pacific Ocular Imaging Society (APOIS) PCV workgroup reached a universal consensus to diagnose PCV without ICGA. The presence of sub-RPE ring-like structures, complex RPE elevation in enface OCT, and sharp-peaked PED in cross-sectional OCT could measure up to a positive predictive value of 0.93^34^. Additionally, OCT has more advantages, such as dye-free procedure, noninvasive, prompt, and safe, which broadens its use.

To date, the pathophysiology and terminology of PCV lesions are controversial. Although its correlations with type 1 choroidal neovasculopathy and pachychoroid spectrum disease were disclosed by the APOIS PCV workshop consensus^34^, the clinical presentation and prognosis still varied immensely. Many classification systems have been attempted to categorise PCV lesions into clinically relevant groups^10,14,15^. In a novel comparative study, Bo et al. observed 43 polypoidal lesions through ICGA, which all corresponded to the “clusters of tangled vasculature” on OCTA^32^. In addition, all polypoidal lesions decreased in size and complexity after anti-VEGF treatment. These thin-walled, tangled vasculature, which stemmed from the BVN, were assumed to potentially account for the clinical features of polypoidal lesions in PCV^32^. Therefore, the authors speculated that BVN patterns, which are the origins of polypoidal lesions, act as potent indicators of disease behaviour and natural course.

In our current study, we divided PCV lesions into three BVN types based on their morphological patterns with clinical relevance. Patients with type 1 BVN had the worst vision at baseline, and the trend persisted until 12 months post-treatment (Fig. 4a, *p* < 0.05 at baseline, 6- and 12-months post-treatment). Furthermore, type 3 BVN encompassed the smallest BVN area among the three types and was thus associated with the most favourable visual outcome after treatment^27^.

Considering the biomarkers (SFCT, CRT, SRF, RPED, and IRC) on OCT that aided in the prediction of the treatment outcome, most of our experience stemmed from the neovascular AMD studies^35,36^. Hata et al.^37^ disclosed that pachychoroidal neovasculopathy was associated with decreased VEGF level in the aqueous and better anatomical outcomes compared with neovascular AMD. Hereby, we found that type 3 BVN had significantly thick SFCT (*p* < 0.001) and less, although not significant, pathologic fluid compartment retention (SRF, RPED, and IRC) at baseline. Multivariate linear regression analysis further confirmed the influences of BVN morphology and IRC on baseline VA in our current study.

Tan’s classification system for PCV focused on the BVN characteristics in ICGA + FA and was first proposed in 2014^17^. The application and prediction of visual prognosis had been constantly verified in several studies^38,39^. In our study, the authors found a significantly high correlation between the two BVN classification systems (*p* = 0.004, Table 2). The result was not so surprising because both systems targeted BVN characteristics, which were regarded as the major pathological features in PCV lesions.

The possible explanations for the worst vision and treatment outcomes in type 1 (trunk form) BVN included the following two aspects. First, the authors noted that trunk form BVN encompassed the largest area over macula under OCTA and foveal involvement was almost inevitable in these cases (Table 1, Fig. 1), and thus posed greater impacts on vision. Second, according to Tan’s proposal, significant and obvious leakage from type C BVN also contributed to less favourable outcomes. Our study also showed high correlation between type 1 (trunk form) BVN and Tan’s type C BVN (Table 2, Fig. 1). On the other hand, our study also demonstrated that visual improvements are more significant in type 2 rather than in type 3 BVN at 12 months post-treatment (Fig. 4a). Patients with type 3 BVN had better BCVA at initial presentation, and thus “ceiling effects” might be subsequently encountered as previous literature speculated^39^. The final BCVA at 12 months post-treatment was significantly related to the BVN types and the presence of IRC. Notably, the SFCT at 12 months post-treatment showed borderline significant effect on the final BCVA (*p* = 0.064, Table 3).

The major limitations of the present study are the nature of the retrospective study design and small case number. The patients in our cohort study were enrolled in 2015 to 2017, when intravitreal anti-VEGF monotherapy was still less frequently adopted as a potent treatment option for PCV. To obey the predetermined study protocols and IRB, the authors thus mainly focused on patients who underwent the combination therapy of PDT and anti-VEGF injections to ensure study population homogeneity. Furthermore, we only included patients with polyps within a 3 × 3 mm central macular area. The aforementioned factors led to certain selection bias. Additional studies with larger population cohorts including both combination therapy and anti-VEGF monotherapy along with prospective design are warranted.

In summary, OCTA provided an innovative method of evaluating PCV lesions and their underlying BVN morphology. Moreover, aging is usually accompanied by increased systemic comorbidities, which should be considered during imaging studies. The main advantages of OCTA are its non-invasiveness and lack of systemic toxicity, making it a safe procedure. Through this study, the authors demonstrated that a strong correlation exists in the BVN imaging morphologies between OCTA and ICGA + FA and further supported the notion that BVN plays a major role in the pathophysiology of PCV lesions. Furthermore, we demonstrated that BVN morphology has important clinical implications, which help clinicians successfully predict the final visual prognosis.
